# *Phragmites australis* (Reed) as an Efficient, Eco-Friendly Adsorbent for Brackish Water Pre-Treatment in Reverse Osmosis: A Kinetic Study

**DOI:** 10.3390/molecules26196016

**Published:** 2021-10-03

**Authors:** Abeer El Shahawy, Inas A. Ahmed, Rabab Wagdy, Ahmed H. Ragab, Nasser H. Shalaby

**Affiliations:** 1 Abeer El Shahawy, Department of Civil Engineering, Faculty of Engineering, Suez Canal University, Ismailia 41522, Egypt; 2 Department of Chemistry, Faculty of Science, King Khalid University, Abha 62224, Saudi Arabia; eaahmed@kku.edu.sa (I.A.A.); ahrejab@kku.edu.sa (A.H.R.); 3 Rabab Wagdy, Environmental Engineering Department, Faculty of Engineering, Zagazig University, Zagazig 44519, Egypt; rabab_wagdi@hotmail.com; 4 Nasser H.Shalaby, Egyptian Petroleum Research Research Institute (EPRI) Cairo, Cairo 11727, Egypt; chem.shalaby@gmail.com

**Keywords:** *Phragmites australis* reed, adsorption, brackish water pre-treatment, column technique, kinetic study

## Abstract

A cost-effective adsorbent was prepared by carbonization of pre-treated *Phragmites australis* reed at 500 °C. *Phragmites australis* was characterized using Fourier transform infrared spectroscopy (FTIR), scanning electron microscopy (SEM) with energy dispersive X-ray spectroscopy (EDX), X-ray diffraction (XRD), and Brunauer–Emmett–Teller (BET) surface analyses. XRD of the as-prepared adsorbent exhibited a partially crystalline structure with a specific surface area of 211.6 m^2^/g and an average pore diameter of 4.2 nm. The biosorption potential of novel biosorbent *Phragmites australis* reed was investigated with a batch scale and continuous flow study. The study was conducted at different constraints to obtain optimum pH conditions, adsorbent dose, contact time, agitation speed, and initial TDS concentration. In order to analyze the properties of the procedure and determine the amount of sodium removal, Langmuir, Freundlich, and Dubinin–Radushkevich isotherms were tested. The optimal values of contact time, pH, and adsorbent dose were found to be 150 min, 4, and 10 g/L, respectively, with an agitation speed of 300 rpm at room temperature (27 °C). The three tested isotherms show that the adsorption of Na^+^ onto the prepared adsorbent is a hybrid process from physi- and chemisorption. For industrial application, the adsorbent was tested using the adsorbent column technique. Pseudo-first-order, pseudo-second-order, and diffusion models were connected, and it was discovered that the information fit best to the pseudo-second-arrange active model. According to the intraparticle diffusion model, the mechanism goes through four stages before reaching equilibrium. The periodicity test shows that the adsorption ability of *Phragmites australis* can be recovered by washing with 0.1 M HCl.

## 1. Introduction

Population growth and economic development drive demand for safe and fresh water in arid and semiarid regions worldwide. Due to a lack of fresh water, people have been forced to reuse low-quality water, such as saline, brackish water, and drainage water. However, salinity can harm plants due to osmotic, nutritional, and toxic stresses [1]. The main cation that causes salinity is the sodium cation (Na^+^), and a high concentration of Na^+^ is an effective limiting factor in using saline water [2].

Groundwater is the primary fresh water supply for many countries’ ever-increasing domestic, farming, and manufacturing sectors. The groundwater in certain areas contains a lot of salt in the form of dissolved cations and anions, making it unsuitable for irrigation. As a result, not all of the water available is of appropriate quality [3]. Poor-quality waters threaten soil conditions and the environment when they are not monitored and cumulative salinity and ionic content is not considered. Although seawater has been proven to be a sustainable and good water supply, its salt content prevents human use. While sodium chloride is considerably higher in salt water than other salts, removing only sodium from seawater does not ensure that drinking water is secure [4]. Various desalination technologies, such as simple distillation, reverse osmosis (RO), ion exchange resins, nano-filtration (NF), and electrodialysis (ED), have been applied and attract greater interest as they possess a great worldwide potential for water treatment. Different technologies were used. Although reverse osmosis is the most applicable, there are certain obstacles: the high price of the membrane, the disposal of brine water, the formation of bio-fouling, and high energy consumption due to the need for high pressure [5].

Unlike the previous techniques, the adsorption process needs little energy and can also operate under gravity conditions. As a result, desalination costs can be reduced by pre-treating salt water before starting these processes. In the RO technique, the applied pressure is chosen according to the degree of salinity of raw water, viz. the total dissolved solids (TDS), to reverse the osmotic pressure [6]. Thus, the reduction in TDS by adsorption reduces the required pressure and the desalination costs. As a result, finding low-cost, high-efficiency adsorbents for removing ions from saline water is an important issue [7]. 

Many research papers concerning using worthless materials to prepare carbon-based adsorbents for water treatment were published, such as sewage sludge, peat, activated carbon from biomass waste, etc. [8]. Based on the same considerations, this study will utilize the *Phragmites australis* (reed) that is the most abundant and dangerous in swamp ecosystems as a new worthless biomass by demonstrating its adsorption performance and process for both sodium remediation from aquatic environments (groundwater and brackish water) and the management of reed harvesting from the constructed wetland. 

Low and little accessible research exists on the study of raw *P. australis*, while it offers several advantages such as simple access or low-cost manufacturing. Furthermore, no reports have yet been made on reed biomass as a sodium biosorbent material, which induces the need for the prospective use of raw *P. australis* in future investigations. The insertion of salt adsorption as a pre-treatment in RO techniques has not been discussed before, which indicates that the current study may be the first exposed to this application. The fundamental aim of this work was to assess the viability of carbonized *P. australis* as a low-cost biosorbent and test its maximum adsorbent efficiency in sodium chloride removal from wells and groundwater.

The following operational parameters were examined to optimize the batch adsorption process at an ambient temperature to achieve the following effects: starting pH of the solution, adsorbing dosage, contact duration, agitation speed, and initial pollutant concentration. The adsorbing material and the efficiency of the adsorption process at different conditions were investigated using Fourier transform infrared spectroscopy (FTIR), scanning electron microscopy (SEM) with energy dispersive X-ray spectroscopy (EDX), surface area (BET), X-ray diffraction (XRD) and atomic absorption of used brackish water constituents. The experimental isotherm and kinetics obtained were adapted to empirical isotherms, kinetic models, and diffusion models. Finally, the best operating parameters were obtained using the column mode on actual groundwater from wells.

## 2. Materials and Methods

### 2.1. Collection and Composition of Brackish Water

Different samples of soil brackish water were drawn from a well in the wild desert of El Natrun, province of Beheira, Egypt. Table 1 lists the key properties of brackish water. All analyses were conducted using standard water analysis procedures. Total dissolved solids (TDS) were determined by the gravimetric technique from the filtrate. Sodium chloride was calculated according to conventional water testing methodologies. The pH of raw water (under examination) was adjusted with NaOH (0.1 M) or HCl (0.1 M) to the desired value. All chemicals used were of an analytical quality and were bought from local sources in Egypt. The standard Sieves Dual Manufacturing Co., USA, was used to sieve the fragmented adsorbent.

### 2.2. Adsorbent Preparation

Samples of *Phragmites australis* were gathered at El-Agoa Canal in Egypt’s Nile Delta region, Zagazig City. The collected plants (leaves and stems) were dissected, soaked in 1% HCl for 48 h at 60 °C, and then washed with de-ionized water to remove any debris adhering to plant components. Then, the plants were placed into a muffle oven (SHIMADEN, Tokyo, Japan) at 500 °C for 30 min to obtain the oven-dried and burnt weight. The seedling samples with a leaf weight to stem ratio of 1/1 *m/m* were finally crushed, grinded and sieved using analytical sieves to determine the optimal particle size. Table 2 illustrates the constitutional features of the reed being used [9].

### 2.3. Optimization of the Adsorption Parameters

The initial part of this study was designed to optimize the conditions that affect the adsorption efficiency. The brackish water was prepared in a laboratory using NaCl as the major solute in natural brackish water. This involved the effect of pH (from 2 to 10), the adsorbent dose (from 0.20 to 10.00 g/L), contact time (from 30 to 180 min), agitation speed (from 100 to 300 rpm), and initial concentration *C_o_* of the adsorbate represented by the total dissolved solids (TDS) in ppm. All experiments were performed in triplicate at the ambient temperature of 27 ± 3 °C and in a constant volume of 100 mL prepared brackish water (dissolving NaCl in distilled water). After optimizing the operational pH, contact time, adsorbent dose, and agitation speed, the optimal values were used to investigate the effect of initial TDS concentration in the range of 1000 to 2000 ppm on the adsorption capacity of the as-prepared adsorbent. For the recovery of the used adsorbent, a 0.4 µm microfiltration membrane filter was used. The sodium adsorption capacity *q_e_* (mg/g) and removal efficiency (*R* %) was calculated using Equations (1) and (2), respectively [10]: 

Adsorption Capacity:

(1) $$q_{e}\left(mg/g\right)=\frac{(C_{o}-C_{e\;})\;V}{W}\;$$

Removal efficiency:

(2) $$R\left(\%\right)=\frac{\left(C_{o}-C_{e\;}\right)}{C_{o}}\;*100$$

*C_o_* is the initial concentration of Na^+^ in ppm, *C_e_* is the remained concentration at equilibrium in ppm, *W* is the sorbent weight in grams, and *V* is the volume of Na^+^ solution in liters.

Finally, all the obtained optimal values were applied in natural brackish water treatment.

### 2.4. Testing Procedures

#### 2.4.1. Investigation of the As-Prepared Adsorbent 

The textural examination of the adsorbent is performed using NOVA 3200 equipment (Quantachrome Instruments, Florida, USA) for nitrogen adsorption–desorption isotherms at 196 °C. For the degassing of the surface, samples were processed at 150 °C for 2 h under vacuum (10^−4^ Torr). The surface area (S_BET_) of the adsorption isotherm branch was estimated using the BET equation. The Barrett, Joyner, and Halenda (BJH) technique was employed to distribute the pores of the branch of the desorption of isotherms. An X-ray diffractometer, PANalytical Model X, pert PRO, equipped with Cu Kά radiations (*k* = 1.5418 A˚), scanned at 0.3 min^−1^, was used to conduct the XRD study. Before and after the sorption process, the surface for the adsorbent was scanned and imaged using a JEOL JSM-6510LV energy-dispersive X-ray spectroscopy scanning electron microscope (EDX SEM) (Jeol, Tokyo, Japan). A Fourier transform infrared (FT-IR) spectrometer (JASCO 4100, Easton, USA) was employed to identify important functional groups on adsorbent surfaces in a wave range of 400–4000 cm^−1^.

#### 2.4.2. Analysis of Raw and Treated Water

The pH values were measured using an AD1000 pH meter (Adwa instruments, Szeged, Hungary). In addition, sodium ion concentrations in crude and processed water were evaluated by atomic absorption spectrometry (AAS) utilizing ZEEnit 700P flame atomic absorption (Analytic Jena GmbH, Jena, Germany). 

## 3. Results and Discussion

### 3.1. Characterization Studies of Fresh and Exploited Adsorbent

#### 3.1.1. FTIR Spectroscopy

**Figure 1:**
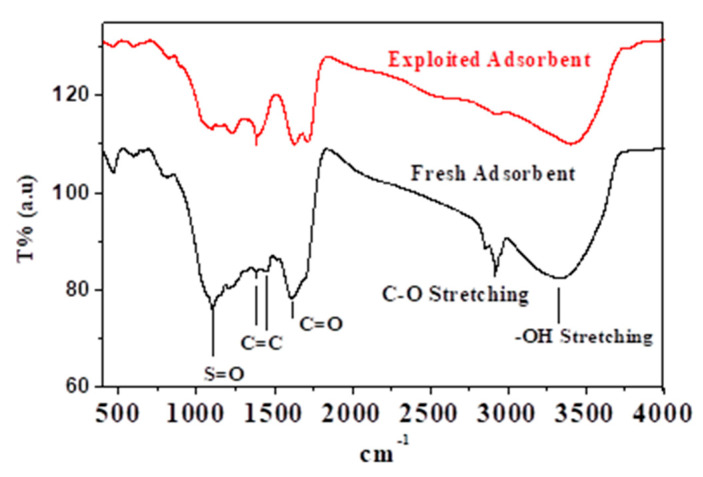
IR spectra of fresh and adsorbent.

Figure 1 depicts the IR spectra of fresh and adsorbent. Different functional groups were identified on the adsorbent surface, as mentioned in the figure. The reduction in the peaks’ intensity and the absence of the peaks in the spectrum of the adsorbent can be used to see the effect of Na^+^ adsorption by various functional groups. The bonding can explain the absence of the C-O peak with Na^+^. As a result, it was hypothesized that the Na^+^ ion could be chemically adsorbed on the biosorbent. Conversely, the reduction in the peak intensity refers to the physisorption of Na^+^, viz. the Na^+^ may be chemically and physically adsorbed. 

#### 3.1.2. Scanning Electron Microscopy (SEM) and Elemental Mapping

**Figure 2:**
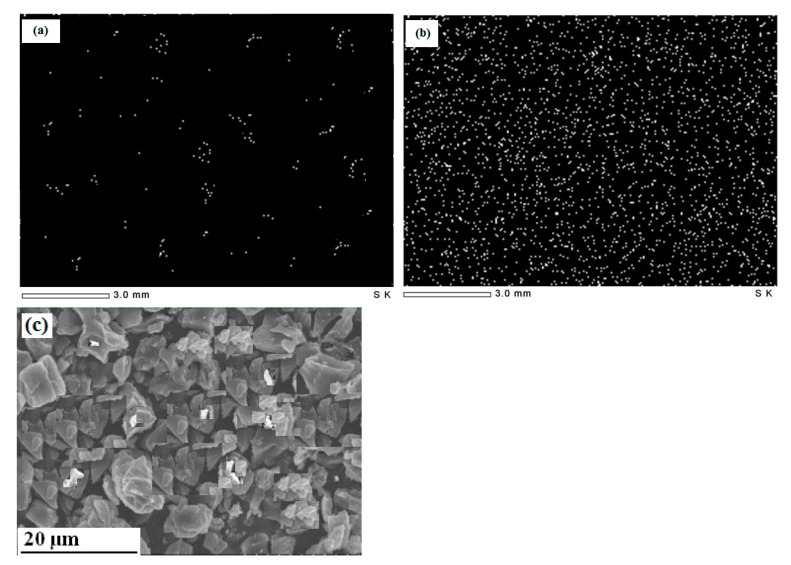
Elemental mapping for cations in: (**a**) fresh adsorbent, (**b**) adsorbent, and (**c**) SEM image of fresh adsorbent.

As shown in Figure 2a,b, the fresh adsorbent contains a few cations that may be attributed to cations embedded in tissues and could not be removed by acid leaching. The elemental mapping of the adsorbent (employed at the optimal values of the dependent parameters) shows densely adsorbed cations with uniform distribution, indicating the homogeneous distribution of active adsorption sites. Figure 2c illustrates the SEM image of the adsorbent surface where certain salt particles can be identified, representing the remaining salts as detected by elemental mapping. The adsorbent surface is full of cavities in the range of 3–5 μm in a slit-shaped structure. 

#### 3.1.3. XRD Results

**Figure 3:**
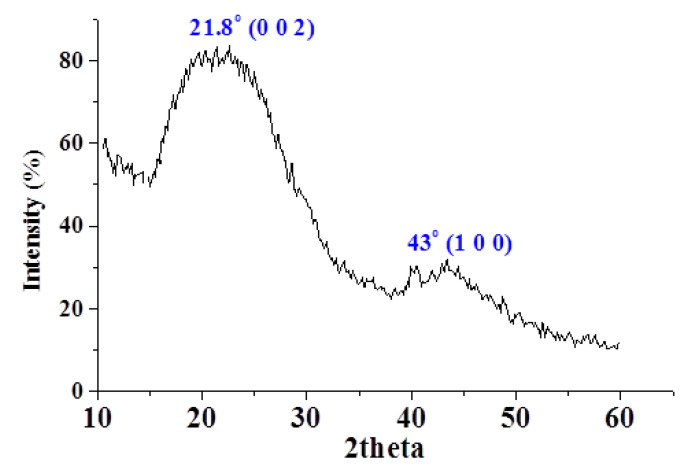
X-ray diffraction pattern of the fresh adsorbent.

Figure 3 illustrates the X-ray powder diffractogram of the as-prepared adsorbent. The spectrum exhibits large diffraction peaks, revealing that the structure is mainly amorphous. The main peak with low broadening, which indicates an increasing regularity of the crystalline structure, is centered at 2Ɵ of 21.8° and assigned to the (0 0 2) plane. The broad peak with low intensity is centered at 2Ɵ of 43° and assigned to the (1 0 0) plane. 

#### 3.1.4. BET Surface Analysis

**Figure 4:**
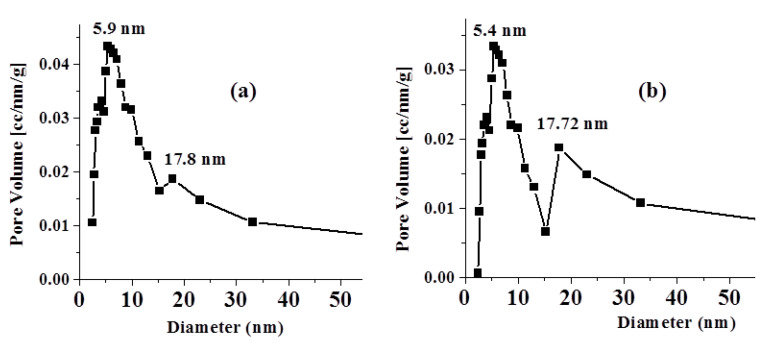
PSD curve of: (**a**) fresh adsorbent; (**b**) adsorbent.

Figure 4 displays the pore size distribution curve (PSD) of fresh and adsorbent. The PSD curves show bimodal pore size distribution curves with two interconnected ranges of pore diameters. The first range with a high population and a most frequent hydraulic diameter of ~5.9 nm starts from 2.45 to 15.26 nm for the fresh adsorbent. The second range with a low population is centered at a hydraulic diameter of 17.8 nm. 

Table 3 depicts the surface parameters of fresh and adsorbent derived from adsorption–desorption isotherms, including specific surface area (S_BET_, m^2^/g), average pore volume (V_P_^0.95^ mL/g) estimated at P/P_o_ of 0.95, and average pore diameter (D_P_^CP^) assuming the cylindrical pore (CP) model. As seen in Table 3, the adsorption of Na^+^ onto the adsorbent surface, however, had only slight effects on the surface area and pore dimensions. Thus, the reduction in surface parameters confirms that the adsorption of Na^+^ most likely occurs on the wall of the pores alongside the outer surface. 

### 3.2. Effect of pH

**Figure 5:**
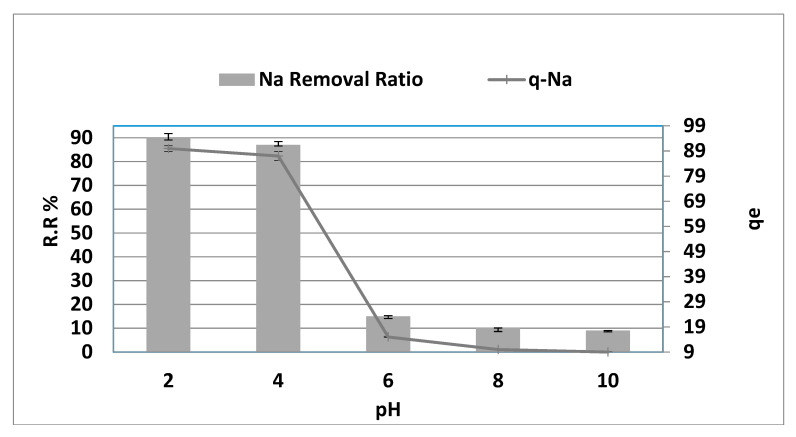
pH effect on NaCl adsorption removal and capacity at adsorbent dosage = 8 g/0.1 L, duration = 150 min, agitation rate = 300 rpm at 270 °C.

The surface load of the AC fluctuates with medium acidity, which affects adsorption. As indicated in Figure 5, the pH reduction from 6.00 to 2.00 improves sodium extraction by between 15.00 and 90.00 percent. In comparison, the pH increase from 6.00 to 10.00 shows decreased sodium extraction by between 15.00 to 9.00 percent (r = −0.893, *p* = 0.042, respectively). Thus, a reduction in pH made the adsorption balance higher, while *q_e_* showed a substantial increase from 14.43 to 93.78 mg·g^−1^ for sodium with a fall in pH to between 6.00 and 2.00. While the pH climbed to between 6.0 and 10.0, *q_e_* declined from 14.43 to 8.78 mg·g^−1^ and vice versa (r = −0.893, *p* = 0.042). Thus, metal ion removal from the solution through contact with a solid phase can be an ion-exchange mechanism in which metal ions replace hydrogen ions on the solid, as observed in the experimental results. Furthermore, at a low pH, the negative sites on the carbon surface are protonated, increasing the surface exchangeable H^+^ to enhance adsorption through cation exchange between Na^+^ and H^+^ on the surface [11]. In contrast, the alkaline medium increases the O content on the carbon surface, making the carbon surface more polar and reducing the adsorption capacity.

### 3.3. The Effect of Adsorbent Dosage

**Figure 6:**
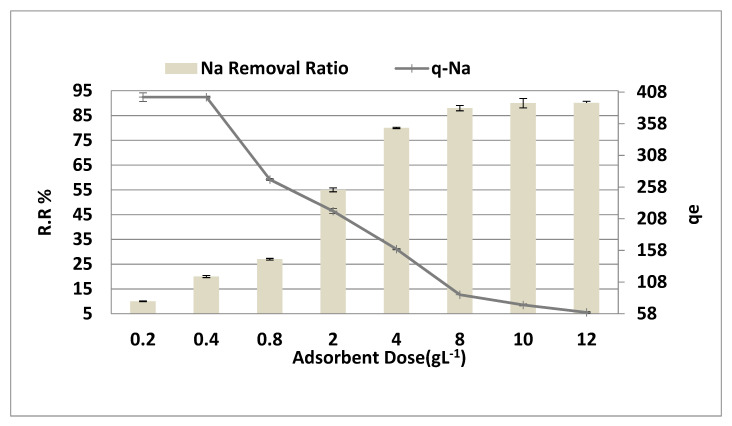
Adsorbent dose effect on the adsorption removal and capacity of NaCl, time = 150 min, pH = 4, and agitation speed = 300 rpm.

Figure 6 shows the adsorbent dose effect at a Na^+^ concentration of 8000 ppm, a time of contact = 150 min, pH = 4, and agitated speed = 300 rpm. As demonstrated in Figure 6, the sodium removal steadily increased from 10% to 90% by increasing the adsorbent dosage by increasing from 0.2 to 10 g·L^−1^ (r = 0.902, *p* = 0.006), which may be attributed to the increase in the available surface area and the active adsorption sites. When the adsorbent dose increased from 10 to 12 g·L^−1^, no significant increase or steady state of adsorption were observed. The adsorption equilibrium is reached by increasing the adsorbent to more than 10 g·L^−1^, and the adsorbent dose becomes insignificant.

Conversely, increasing the adsorbent dose had a negative effect on the adsorption capacity (*q_e_* mg/g). Higher adsorbent dosages have been found to result in a higher percentage of removals. However, the diffusion decreases as adsorbent amounts increase, as several variables include a decreased solvent ratio, interference between binding sites, and electrostatic interactions [12].

### 3.4. The Effect of Contact Time

**Figure 7:**
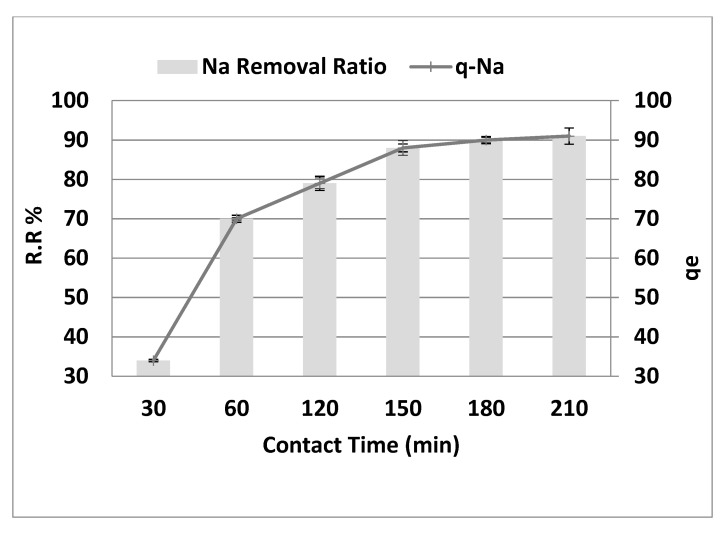
Effect of contact time on the removal efficiency and the adsorption capacity of NaCl, adsorbent dose = 10 g·L^−1^, pH = 4, and agitation speed = 300 rpm.

Figure 7 depicts the effect of contact time on the behavior of Na^+^ adsorption onto the adsorbent surface at the optimum values of the other dependent factors (pH = 4, adsorbent dose 10 g·L^−1^, initial concentration = 8000 mg·L^−1^, and agitation speed = 300 rpm). As shown in Figure 7, the sodium removal gradually increased from 34% to 91%, increasing contact time from 30 to 210 min (r = 0.887, *p* = 0.019). Similarly, *q_e_* reached 91 mg·g^−1^ after 210 min (r = 0.887, *p* = 0.019). However, no substantial removal elevation was noted with additional contact time increasing from 150 to 210 min. The *q_e_* was also steady when contact time was raised from 150 to 210 min. The amount of sodium adsorbed increases as the contact time extends to 150 min after equilibrium. The efficiency of salt removal in the early stage (<150 min) increased considerably due to the active binding sites on the fresh adsorbent. However, as time went on, the removal became less efficient due to the slow saturation of the functional binding sites until they were all used up. The first step is thought to lead to surface adsorption, and the second step is believed to lead to intraparticle transport from the bulk fluid to the porous adsorbent’s external surface [13]. This finding agrees with the reported results of other investigators. Adsorption is rapid in the early phases of the contact time and then slows down toward equilibrium due to a high number of vacant surface regions in the original stage that are available for adsorption which slowly become saturated [14]. However, the remaining empty spaces are difficult to fill due to repulsive interactions between solute molecules during the solid and bulk phases.

### 3.5. The Effect of Agitation Speed

**Figure 8:**
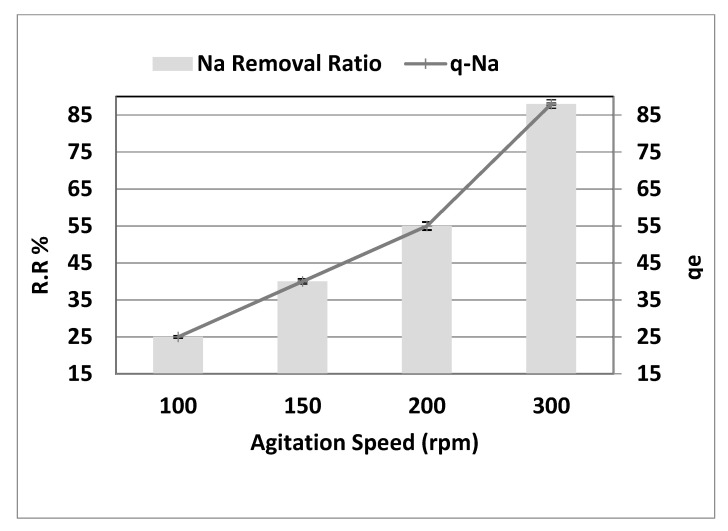
Effect of agitation speed on the removal efficiency and the adsorption capacity of NaCl, adsorbent dose = 10 g·L^−1^, pH = 4, and contact time = 150 min.

Figure 8 depicts the influence of stirring rate on sodium sorption in the range of 100–300 rpm at a pH of 4, starting concentration (*C_o_*) of 8000 mg·L^−1^, 10 g·L^−1^ adsorbent dosage, and a contact period of 150 min. The impact of the stirring rate is as follows: the results shown in Figure 8 show that the salt removal rate rises with an increase in agitation speed. The most remarkable effect of removal (25%, 40%, 55%, and 88%) was observed at a rate of 100, 150, 200, and 300 rpm, with (r = 1.00, *p* = 0.000). In addition, *q_e_* showed a substantial increase of 25 to 88 mg·g^−1^ (r = 1.00, *p* = 0.000) with a rise in agitation speed between 100 and 300 rpm due to the dispersion of adsorbent molecules. Because the mass transfer governs the adsorption process, the procedure regulates the liquid side mass transfer resistance. As a result, as the bulk moves, the adsorption rate increases. Conversely, strong stirring results in further adsorption–desorption on the adsorbent surface, leading to adsorbate–adsorbent bonding to likely be broken. It is widely known that the adsorption rate is controlled based on the degree of agitation of the fluid particle system, either by film diffusion or by pore diffusion [15].

The particle’s film is thicker at a lower agitation speed, and the film diffusion appears to be a rate-limiting phase. Due to that, the adsorption kinetics are impacted by poor adsorption mass transfer to the interior surface of the particle. In contrast, the diffusion of the film at high agitation speeds increases to a maximum value when the pore diffusion becomes the control rate step [16].

### 3.6. Initial Concentration Effect

**Figure 9:**
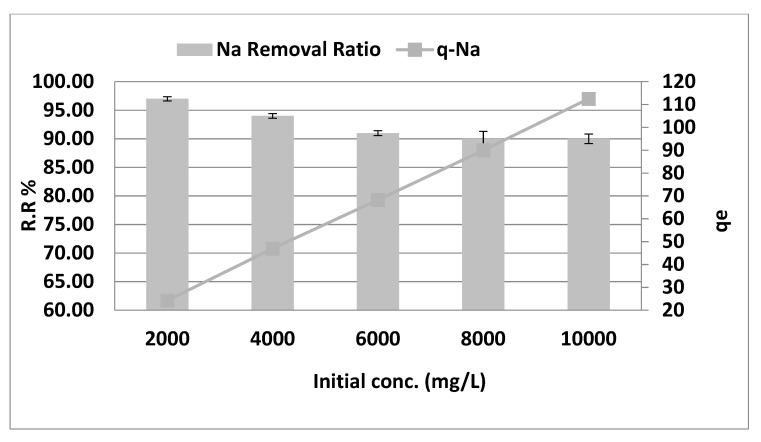
Effect of initial conc. of NaCl on the removal efficiency and the adsorption capacity of NaCl at adsorbent dose = 8 g·L^−1^, pH = 4, time = 150 min, and agitation speed = 300 rpm at 27 °C.

The initial concentration of Na^+^ was tested in the 2000–10,000 mg·L^−1^ range under the pre-optimized dependent parameters, i.e., pH = 4, a 10 g·L^−1^ adsorbent dose, and a contact period of 150 min. As demonstrated in Figure 9, the increase in the initial sodium level from 2000 to 10,000 mg·L^−1^ leads to a decline in the percentage of NaCl removal from 97% to 90% (depending on the initial concentration of each batch) (r = −0.933, *p* = 0.02). Meanwhile, *q_e_* rose from 24.25 to 112.50 mg·g^−1^ (r = 1.00, *p* = 0.00). The greater starting sodium concentration with the constant quantity of adsorbent results in a greater sodium level in the solution, leading to increased sodium adsorption by the adsorbent. The increase in adsorption with the rise in sodium is due to a large mass transfer driving force [17]. When the initial concentration increases within the limits of adsorption capacity or below, both the removal efficiency and the adsorption capacity increase, which was confirmed by the results observed when the initial concentration varied between 20 and 100 mg·L^−1^ (under the previous values of other dependent parameters). The percentage removal grew linearly such that the removal of NaCl ranged from 50% to 92%. (r = 0.989, *p* = 0.096) and *q_e_* increased from 0.125 to 1.15 mg·g^−1^ with (r = 0.99, *p* = 0.092). 

### 3.7. Adsorption Kinetics and Isotherm Models

Kinetic models represent the interaction between pollutant adsorbate molecules or ions on the adsorbent surface and active sites (pseudo-first order, pseudo-second order). These models do not consider diffusion, yet it is known that intraparticle diffusion may impact kinematic observations. Models of diffusion (film–porous diffusion, film–surface diffusion, and film–parallel pores and surface diffusion models) presume that interplay between pollutant adsorbates and active sites is instantaneous concerning diffusion stages, hence governing the rate of diffusion. The following sections provide brief descriptions of various models. The potential application of pseudo-first- and -second-order models was assessed for bio-sorption kinetics [18,19,20]. 

Kinetic models simulate interactions between adsorbed pollutant molecules or ions and active surface areas (pseudo-first order, pseudo-second order). These models take no account of diffusion; however, it is known that intraparticle diffusion may alter kinetic data. Models for diffusion (film–pore diffusion, film–surface diffusion, film–parallel polar model, surface diffusion model) presume that interaction between adsorbed pollutants and active sites is instantaneous compared to diffusion steps, affecting the total rate [21]. In the following sections, a short explanation of the different models is given. The potential application of pseudo-first- and -second-order models was studied for Biosorption Kinetics. NaCl adsorption in the created biosorbent was examined in the Na^+^ concentration by Freundlich, Langmuir, and Dubinin–Radushkevich (D–R) methods. Langmuir isotherm is a quantitative description of creating a monolayer adsorbate on the surface of the adsorbent not followed by additional adsorption. The Langmuir method therefore defines the distribution of metal ions between the solid and liquid phases in equilibrium. The Langmuir isotherm largely matches the adsorption on a surface with a limited number of the same sites. The model assumes homogeneous adsorption energies on the surface and no transmigration of adsorbates on the surface plane [22]. 

#### 3.7.1. The Adsorption Kinetic Parameters

The pseudo-first-order and pseudo-second-order models were used to analyze the experimental data (refer to Supplementary Materials). 

The obtained parameters are shown in Table 4, and the experimental results clearly demonstrate that the pseudo-second-order model with R^2^ = 0.98 suits the experimental results. In addition, the theoretical values of *q_e_* in this model are similar to the observed values. These findings suggest that a pseudo-second-order model can adequately explain adsorption kinetics. 

The findings of this research correspond with the conclusions of the research of Rostamian et al. [23]; they studied the sodium (Na^+^) sorption capacity of active carbon generated under physical activation by steam, potassium-hydroxide (KOH) chemical activation, and KOH-damp physiochemical activation. In adsorption models, carbohydrates obtain the pseudo-first-order (R^2^ = 0.995) and intraparticle models (R^2^ = 0.993, *K*_1_ = 3.84 mg·g^−1^ min^−0.5^, *C* = 10.07 mg·g^−1^). These data show that the physical adsorption of macropores, mesopores, and micropores dominate the activated carbon. 

The results of this study were based on a study by Santiago et al. [24], who investigated how the adsorption of co-produced water from Bowen Basin (QLD, Australia), using natural, untreated zeolite, could adsorb Na+ ions of 16.16 mEq/100 g and NH^4+^-treated zeolite using a 1.0 M ammonium acetate (NH_4_C_2_H_3_O_2_) solution. The theoretical capacity of exchange for natural zeolites was 154 mEq/100 g. The adsorption of Na^+^ ions in natural zeolite has attained a capacity of 14.34 mEq/100 g after 720 min and a rate of adsorption indicated by the *k*_2_ = 0.002 mEq/100 g min pseudo-second-order kinetic model. The homoionic treatment of zeolite materials has increased the Na+ adsorption rate and capacity. The highest sodium adsorption and rate in NH^4+^ occurred after 720 min, computed using a pseudo-second-order kinetic model, i.e., *q_e_* = 38.28 mEq/100 g and *k*_2_ = 0.002 mEq/100 g min^−1^. Thus, chemical sorption is the limiting step of the rate, i.e., chemisorption between Na^+^ and zeolite. 

The kinetic adsorption data from removing sodium ions from aqueous solutions by adsorption in amorphous carbon thin film (ACTF) were investigated for batch mode by Fathy et al. [4]. The ACTF has a maximum sodium adsorption capacity of 107, 120, and 135 mg·g^−1^ at 35, 45, and 650 °C. The adsorption of Na^+^ on the ACTF examined is a highly complicated and spontaneous process, suggesting that both physisorption processes and chemisorption processes are involved. The pseudo-second-order parameters are most suited for explaining the contact time research findings. The particular form prevalent in the pseudo-second-order kinetics when metal ions are taken away from carbonate materials indicates that both Na^+^ and adsorbent (ACTF)-supportive concentrations must decide the stage’s adsorption process.

Furthermore, the slower absorption rate in ACTF indicates the adsorption of sodium ions with a more considerable energy barrier. It may be similar to the operating design and surface complexion. The findings showed improved conformity with the pseudo-second-order model, and the regression coefficients for the linear compartments were more than 0.999. The kinetics sorption corresponded with the pseudo-second-order model reported by El Shahawy and Heikal [25] for total dissolved solids removal. The pseudo-second-order parameters were 0.000534 gm g^−1^ min^−1^ and 59.5238 mg·g^−1^ for *k*_2_ and *q_e_*, respectively. 

#### 3.7.2. Adsorption Isotherms

The adsorption of NaCl on the prepared adsorbent at equilibrium as a function of Na^+^ initial concentration was investigated using Freundlich, Langmuir, and Dubinin–Radushkevich (D–R) isotherms. The Langmuir isotherm is a quantitative description of the formation of a monolayer adsorbate on the adsorbent’s outer surface, followed by no further adsorption. Consequently, the Langmuir isotherm describes the distribution of metal ions in an equilibrium between the solid and liquid phases. The Langmuir isotherm is mostly suitable for adsorption on a surface with a finite number of identical locations. The model assumes homogeneous adsorption energies on the surface and no transmigration of the adsorbate in the surface plane [26,27] (refer to Supplementary Material). 

The 1/n was from 0.00 to 1.00, indicating that sodium biosorption on *Phragmites australis* was conducive to the conditions under study. The functional site distribution or other causes for this could be that adsorbent–adsorbate interactions decrease with an elevation in surface density attributed to 1/n < 1.00. The friendly model posits that adsorption may take place on several levels to prevent the occurrence of saturation.

Fathy et al. [4] examined the adsorption properties of sodium ion removal in batch mode, using adsorption of amorphous thin-carbon film (ACTF). The Na^+^ experimental adsorption data on ACTF follow the isotherms of Freundlich adsorption. An adsorption isotherm for the *K_F_*, 1/n, and R^2^ model Freundlich coefficients (4.055, 0.239, 0.992) were used by *Phragmites australis* for ACTF, exploring the higher monolayer biosorption. The Dubinin–Radushkevich isotherm (D–R) is commonly used to determine if adsorption is physisorption or chemisorption. This model’s linear form is as follows [28]:

(3) $$\ln q_{e}=\ln q_{m}-\beta \varepsilon^{2}\;$$

*q_m_* is the theoretical isotherm saturation capacity (mg/g), *Ԑ* is Polanyi’s potential, and *β* is related to the adsorption energy for each mole of the DBT (mol^2^·kJ^−2^). The power accompanied by the traveling of the adsorbate molecule from the bulk to the adsorbent surface (KJ/mole) is given by [15]:

(4) $$E=\sqrt{2\beta }\;$$

The value of *E* is an index in the adsorption type; *E* ˂ 8 Kj mol^−1^ indicates a physisorption process, but when *E* ˃ 8 Kj mol^−1^, the adsorption is a chemisorption process. The estimated parameters from the tested models are presented in Table 5. As shown, the Freundlich isotherm fits the experimental results with R^2^ = 0.987, which indicates a multilayer adsorption mechanism. The calculated value for 1/n (0.525) refers to the heterogeneous nature of the adsorbent surface. The Langmuir isotherm is second only to Freundlich in terms of fitness with the practical results with R^2^ of 0.884, and lastly, the D–R isotherm has an R^2^ of 0.753. The tested models refer to hybrid adsorption processes, viz. both physi- and chemisorption occur. Adsorption on the microcrystallite planes mostly results from van der Waals forces. Conversely, adsorption at the edges of the microcrystallite occurs due to chemical bonding. The higher *E* value (26.5 KJ mol^−1^) may be attributed to the strong electrostatic adsorption of Na^+^. 

Therefore, by comparison, the order of the isotherm best fits of the four sets of experimental data in this study is Freundlich > Langmuir > Dubinin–Radushkevich.

#### 3.7.3. Adsorption Mechanism

Three sequential stages may occur in the adsorption process for sodium removal from an aquatic medium: (i) external mass transfer bulk diffusion; (ii) intraparticle diffusion film diffusion; and (iii) adsorption at active areas. It has been stated in the literature that the first step might be “neglected” by the adequate stirring speed with mechanical augmentation. Intraparticle diffusion involves the effective diffusion of pore volume, surface diffusion, or combining the two processes [29]. The effective diffusion of pores shows adsorbate (ions) movement into the particle during the liquid phase [25]. Surface diffusion is associated with transporting the adsorbate from higher energy sites to locations of less energy on the surface of the adsorbent particles. The adsorption data obtained were examined using the intraparticle diffusion model of Weber and Morris. In this model, the starting rate of intraparticle diffusion is established using the following equation [29]:

(5) $$q_{t}=k_{i}t^{0.5}+C\;$$

where *k_i_* is the intraparticle diffusion step constant (mg/g·min^0.5^), and *C* (mg/g) is the constant parameter; the boundary layer effect indicates where *C* exhibits increasing values as it grows. From Equation (5), linear results are produced when *q_t_* is plotted against (*t*^0.5^), and if diffusion is not the only rate-limiting process in the intraparticle phase, this line does not pass through the origin.

**Figure 10:**
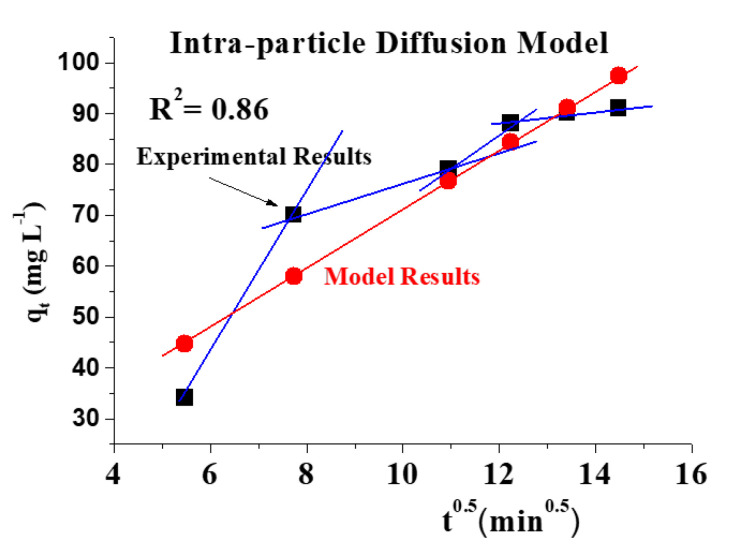
Diffusion figure for adsorption of Na^+^ intraparticle with an initial concentration of 10,000 mg/L at 27 °C, pH = 4 over 500 min.

**Figure 11:**
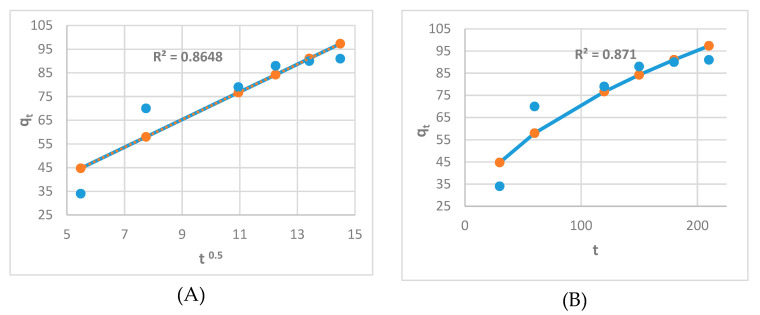
(**A**) linear Intraparticle diffusion kinetic model; (**B**) Nonlinear Intraparticle diffusion kinetic model.

Figure 10 demonstrates that the adsorption of the adsorbent used passes through four phases to achieve equilibrium. Concerning Figure 11A,B, a linear and nonlinear relation exists at all times, although it does not pass through the origin. It shows that intraparticle diffusion was present but is not the sole rate-control step and that there may be additional mechanisms. Both film and pores regulate the mechanism of intraparticle diffusion. The plot of Na^+^ fractional absorption (*q_t_*/*q_e_*) vs. *t*^0.5^ reveals portions that suggest a highly rapid initial stage; then, a sluggish final absorption of Na ions into pores corresponding to the pattern of the intraparticle diffusion model of Weber and Morris occurs.

Film diffusion and pore diffusion were suggested to learn more about the mechanism and the rate control processes impacting the adsorption kinetics. The equation of film diffusion may be represented as (Fathy et al. 2017 [4]):

(6) $$\frac{q_{t}}{q_{e}}=6{\left(\frac{D_{1}}{\pi a^{2}}\right)}^{\;\;\;0.5}t^{0.5}+C\;$$

where a (μm) is the average *Phragmites australis* radius, and *D_1_* is the film diffusion coefficient (μm^2^ S^−1^).

The Na^+^ plots of *q_t_*/*q_e_* vs. t^0.5^ are consistent with diffusion, containing three portions. The diffusion of Na^+^ ions from the exterior surface of carbonized *Phragmites australis* via the boundary layer reveals dominating control. By using the pore diffusion model in comparison, we can characterize the adsorption kinetics. Reichenberg formulated the equation of pore diffusion as follows [4]:

(7) $$\frac{q_{t}}{q_{e}}>0.85,\;Bt=-0.4977-\ln(1-\frac{q_{t}}{q_{e}})$$

(8) $$\frac{q_{t}}{q_{e}}<0.85,\;\;Bt={\left(\sqrt{\pi }-\sqrt{\pi -\left(\frac{\pi^{2}}{3}*\frac{q_{t}}{q_{e}}\right)\;}\right)}^{2}$$

The square root of time (*t*^0.5^) represents the fractional absorption of Na^+^ (*q_t_*/*q_e_*). The plots of fractional uptake of Na^+^ vs. *t*^0.5^ for carbonized *Phragmites australis* showed plots with sections reflecting an early, highly rapid stage followed by a slow final absorption of Na^+^ ions into the pores, which was comparable to the pattern of intraparticle diffusion. The Na^+^ adsorption film diffusion (D_1_) in carbonized *Phragmites australis* are calculated on the slopes of the *q_t_*/*q_e_* plots against *t*^0.5^ and displayed in Table 6. Causes for the greater D_1_ value for carbonized *Phragmites australis* are suggested: repulsion from the positively charged Na^+^ generated by the carbonized *Phragmites australis*/Na^+^ system as it crosses the liquid-layer pH-positioned adsorbent surface, and roughness on the surface of the adsorbent. In the film diffusion coefficient of the 10^−6^–10^−8^ cm^2·^s^−1^ range, Pholosi et al. [30] revealed that film diffusion is active in the adsorption mechanism. In our investigation, the magnitude of the film diffusion coefficient for carbonized *Phragmites australis* ranged from 10^−7^ and showed that film diffusion was active in the Na^+^ adsorption process on carbonized *Phragmites australis*. *B* can be used to calculate the effective pore diffusion coefficient, *D_2_* (μm^2^ S^−1^), from the following equation [31].


(9) $$B=\pi \frac{D_{2}}{r_{2}}$$

In an ideal linear regression (*Bt* vs. *t* plot), the porosity control defines the mass transfer rate while passing through the original fit. Therefore, the conclusion can be drawn that the film diagram or chemical reaction also regulates the adsorption rate. The plot is non-linear or linear only when the intercept differs from zero. As demonstrated in the table, *Bt* vs. *t* for Na^+^ ion adsorption was originally not passed through and displayed a non-linear segment at short adsorption times, further substantiating the above claims that film dissemination or chemical reaction control adsorption during that time [30,31].

### 3.8. Application to the Treatment of Brackish Water

As soon as the adsorption process parameters were optimized using the laboratory-prepared brackish water, the adsorbent was used to treat natural brackish water with chemical analysis, represented in Table 7, under the observed optimal values. From this table, the removal efficiency for the contaminated salts (TDS) is ~79.85%. In water treatment by the RO technique, which is a commonly used technique, the requested pressure to overcome the osmotic pressure is directly proportional to the TDS of raw water. The osmotic pressure is defined as the lowest pressure given to a solution to stop the passage of solvent molecules across a semipermeable barrier. The equation provides the osmotic pressure (osmosis):

(10) π=iCRT 

where ***π*** is the osmotic pressure, **i** is the Van ’t Hoff factor, **C** is the molar concentration of solute in the solution, **R** is the universal gas constant, and T is the temperature. 

According to Equation (11), reducing TDS from 10,000 to 2015 ppm at a constant temperature means reducing the requested pressure to one-fifth. In addition, the removal of TDS of the raw water increases the recovery of the RO plant and increases the life of the used membranes [32].

From Table 7, it can be noticed that the sodium ion is the most concentrated (80%), followed by calcium (5%) and potassium at 3.5%. The removal efficiency of the three cations is 90%, 66.8%, and 70.85%, respectively. In an aqueous medium, the adsorption tendency of a cation depends on its initial concentration *C_o_* and its hydration energy. The increase in initial concentration drives the cations to the adsorbent surface, as discussed in Section 3.6. On the contrary, the rise in hydration energy means more solubility and less tendency for adsorption. The hydration energy depends on the cation radius, where a smaller cation can accommodate a large number of water molecules around it and become hydrated [33]. The radii of three cations, Na^+^, Ca^++^, and K^+^, are 116 pm, 114 pm, and 152 pm, respectively. These radii are accompanied by hydration energies of 51, 311, and 34 Kcal/g ions, respectively. The observed values in Table 7 seem to be in good agreement with the previous studies. In the presence of K^+^, Ca^2+^, and Na^+^, there was no significant adsorption capacity reduction in concentrations of 350, 500, and 8000 mg/L, respectively, because of its affinity with alkaline and alkaline–earth metal ions. The co-existence of ions rarely impacted the elimination of Na^+^ ions. The existence of other co-existing anions shows that there was no substantial adsorption loss for the removal of Na^+^ at concentrations of 10,000, 10,000, and 8000 mg/L, respectively, for SO4^++^, CO^2+^, and Na^+^.

### 3.9. Periodicity

From a practical and economic point of view, the reusability of adsorbents is a fundamental stage to establish its use in water purification technology. Owing to the slow deposition of adsorbate on the adsorbent surface, the adsorbent’s efficiency is progressively diminished, eventually exhausting it. The extent of adsorbent–adsorbate interactions is crucial in the regeneration process [34]. To evaluate the employed adsorbent, 0.1 M HCl was used in the elution of the adsorbate. The chosen HCl solution is due to the smaller size of Cl^−^ ions than that of NO^3−^ and SO_4_^2−^ ions and avoiding the formation of insoluble salts when using sulfuric acid [35]. After elution, the re-generated adsorbent was rinsed thoroughly with de-ionized water to remove any traces of HCl. Then, the adsorption–desorption cycle was repeated six times under optimal conditions using the drawn natural brackish water (TDS = 10,000 ppm). As shown in Table 8, in six cycles, the removal rate of sodium (R%) was slightly decreased. This observed decrease may be linked with the incomplete removal of the adsorbate and/or the loss of the associated adsorbent material during the repeated cycles [36]. 

### 3.10. Industry Application

For application in industry, the adsorbent had to be tested under dynamic conditions using the adsorbent column technique. At room temperature, the adsorption performance of the as-prepared adsorbent was achieved using a continuous flow adsorption experiment in a fixed-bed glass column with an inner diameter of 1.0 cm, a height of 15 cm, and a medium porosity sintered-Pyrex disc at its bottom to prevent material loss. Approximately 100 mL of demineralized water was run through the column before use in the experiments to remove any non-consolidated material and ensure the absence of soluble species. The flow rate was adjusted at 9 mL min^−1^ using Watson Marlow 505 S Peristaltic Pump with an adjustable flow rate between 0.6 microliters and 42 milliliters min^−1^ with a speed range of up to 220 rpm.

**Figure 12:**
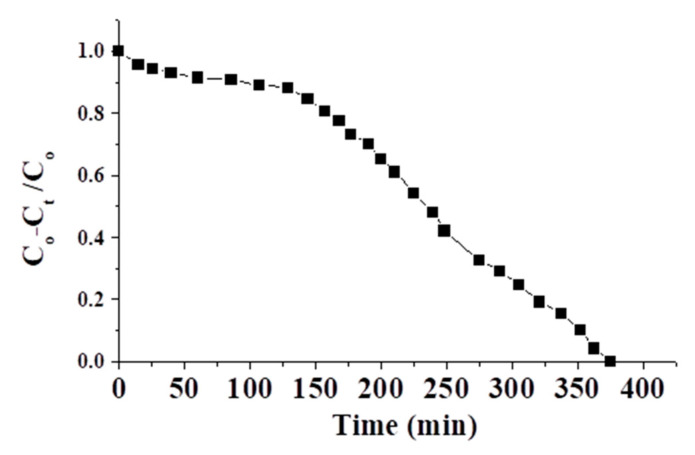
The breakthrough curve for brackish water of TDS = 10,000 ppm.

Figure 12 shows the breakthrough curve of the brackish water of TDS = 10,000 ppm and pH = 5.5 (which can meet the requirements of RO membranes). The flow to the column continued until the effluent salt concentration (TDS) at time t (*C_t_*) reached the influent salt concentration (*C_o_*), viz., *C_o_* − *C_t_*/*C_o_* ≈ 0. At a fixed flow rate, the retained amount of salt in the column (column adsorption capacity in ppm) was calculated graphically by numerically integrating the area under the curve (*C_o_* − *C_t_*)/*C_o_* vs. service time (as shown in Figure 12) using the Origin 5 program. According to the obtained result, the adsorption capacity of the adsorbent was ≈ 57.5 ppm g^−1^. The deviation of this result from the batch results may be linked with the dynamic. This result shows the feasibility of this adsorbent in the industry. 

## 4. Comparison with Other Studies

Table 9 shows that carbonized *P. australis* has better adsorption effectiveness than most adsorbents. Briefly, carbonized *P. australis* is a low-cost sorbent and the plant *P. australis* is very abundant and cheap, the adsorbent manifesting strong adsorption, and solid mechanical stability, indicating that *P. australis* is a solid option for economical feasibility.

## 5. Conclusions 

The removal of Na^+^ as a biosorbent from aqueous solutions using *P. australis* was examined by batch processing. The results show the effect of the experimental conditions on the capacity of sodium adsorption, such as adsorbent doses, pH values, starting levels, and time of contact. The maximum sodium adsorption capacity of 117.68 mg/g was found at pH = 4. Equilibrium studies show that the data are well suited to the Freundlich isotherm based on the correlation coefficient (R^2^ = 0.98). The kinetics of adsorption demonstrated that the linear model of the second-order is best described during adsorption. The sodium desorption investigations have shown the favorable potential of *P. australis* biosorbent regeneration and reuse, the biosorbent utilized in this work with several prospective commercial benefits in the future. The results have shown that *P. australis* is economically attractive for the treatment of wastewater. Reed biomass has been shown to have excellent adsorption performance with high and low sodium levels, simple pre-treatment of the adsorbent material, and ease and high desorption and sorption capacity regeneration feasibility. Because of the availability of reed biomass and constructed wetlands where a mature reed recovery is desired, such features will contribute to future usage as a biosorbent material to remove further pollutants of this unique plant biomass.

## Figures and Tables

**Table 1:** The characteristics of the underground brackish water (pH = 8 at T °C = 24).

Color	Turbidity (NTU)	Conductivity (µs/cm)	ζ- Potential (mv)	BOD (ppm)	COD (ppm)	TDS (ppm)	Na (ppm)	Ca (ppm)	K (ppm)
Light grey	25 ± 2	10,000 ± 5	−25 ± 2	Nil	Nil	10,000 ± 3	8000 ± 2	500 ± 1.5	350 ± 0.5

**Table 2:** Constitutional characteristics of *Phragmites* *australis* (%*w/w*) ^a^ (source from El Shahawy and Heikal, 2018a [9]).

Proximate Analysis (wt%)	Leaves	Stems	Fiber Analysis	Leaves	Stems
**Ash**	4.50 ± 0.02	5.10 ± 0.03	**Cellulose**	39.50 ± 1.75	42.70 ± 1.83
**Moisture**	3.70 ± 0.15	4.20 ± 0.11	**Lignin**	29.69 ± 3.15	27.27 ± 2.38
**Volatile**	42.00 ± 0.23	36.10 ± 0.21	**Hemicellulose**	23.61 ± 0.52	23.73 ± 0.41
**Fixed Carbon**	49.80 ± 0.36	54.60 ± 0.32	**Extractives**	7.20 ± 0.74	6.30 ± 0.89

^a^ All values are the mean ± SD mean for three replicates.

**Table 3:** BET surface area and pore dimensions of fresh and adsorbent.

Sample	S_BET_ (m^2^/g)	D_P_^CP^ (nm)	V_p_^0.95^ (mL/g)
Fresh Adsorbent	211.6 ± 0.17	4.2 ± 0.23	0.215 ± 0.25
Adsorbent	188.5 ± 0.25	3.77 ± 0.34	0.187 ± 0.38

**Table 4:** Detected kinetics parameters for adsorption of Na^+^ at C_o_ = 10,000 mg·L^−1^, 27 °C, pH = 4 for 150 min.

*q_e_* (mg/g) Experimental	Pseudo-First-Order			Pseudo-Second-Order		
	*q_e_* (mg/g)	*K_*1*_* × 10^3^ (min^−1^)	R^2^	*q_e_* (mg/g)	*K_*2*_* × 10^3^ (g·mg^−1^·min^−1^)	R^2^
**112.5**	92.17	0.0187	0.97	**119.8**	0.000139	0.98

**Table 5:** Obtained data from fitting experimental results with the three tested (linear and non-linear) isotherm models.

Langmuir Model	Plotting	*q_o_* (mg·g^−1^)	*K_L_* (L mg^−1^)	*R_L_*	R^2^
$\mathrm{Nonlinear}:\;\;q_{e\;}=q_{o}\frac{K_{L}C_{e}}{1+K_{L}C_{e}}$	$q_{e\;}\;\mathrm{vs}.\;C_{e}$	189.7632	0.001245	0.07–0.29	0.959518
$\mathrm{Linear}:\;\;C_{e}/q_{e}=1/q_{o}K_{L}+C_{e}/q_{o}$	$Ce/q_{e}\;\mathrm{vs}.\;C_{e}$	146.795	0.002228	0.04–0.18	0.884024
**Freundlich model**		**K_f_** **((mg/g)/(mg/L)n)**	**n**		**R^2^**
$\mathrm{Nonlinear}:\;q_{e}=K_{f}C_{e}^{1/n\;}$	$q_{e\;}\;\mathrm{vs}.\;C_{e}$	1.728578	1.674918		0.98078
$\mathrm{Linear}:\;\ln q_{e}=\ln K_{F}+\frac{1}{n}\ln C_{e}^{\;}$	$\ln q_{e}\;\mathrm{vs}.\;\ln C_{e}^{\;}$	2.729497	1.90501		0.987677
**Dubinin–Radushkevich model**		***q_m_***	***β***	***E* (kJ·mol^−1^)**	**R^2^**
$\mathrm{Nonlinear}:\;q_{e}=q_{m}\;exp^{-\beta \varepsilon^{2}}$	$q_{e}\;\mathrm{vs}.\;\varepsilon^{2}$	83.39953	0.000996	−22.401	0.596461
$\mathrm{Linear}:\;\ln q_{e}=\ln q_{m}-\beta \varepsilon^{2}$	$\ln q_{e}\;\mathrm{vs}.\;\varepsilon^{2}$	77.95046	0.000712	−26.4994	0.75261

**Table 6:** Obtained data from fitting experimental results with the three tested models.

The Pseudo-First-Order Model	Plotting	*k_1_* min^−1^	*q_e_* mg·g^−1^	R^2^
$\mathrm{Nonlinear}:\;q_{t}=q_{e}\;(1-e^{-k_{1}t})$	$q_{t\;}\;\mathrm{vs}.\;t$	0.018782	92.7422	0.957782
$\mathrm{Linear}:\log\left(q_{e}-q_{t}\right)=\log\left(q_{e}\right)-k_{1}t/\ln10$	$\log\left(q_{e}-q_{t}\right)\mathrm{vs}.\;t$	0.018677	92.17512	0.972588
**The Pseudo-Second-Order Model**	**Plotting**	***k*_2_** g mg^−1^ min^−1^	***q_e_*** mg·g^−1^	**R^2^**
$\mathrm{Nonlinear}:\;q_{t}=k_{2}q_{e}^{2}t/(1+k_{2}q_{e}t)$	$q_{t\;}\;\mathrm{vs}.\;t$	0.000154	117.8253	0.942221
$\mathrm{Linear}:\;t/q_{t}=1/(k_{2}q_{e}^{2})+t/q_{e}\;$	$t/q_{t}\;\mathrm{vs}.\;t$	0.000139	119.771	0.974754
**Intraparticle Diffusion Model**	**Plotting**	***k_1_*** mg·g^−1^ min^−0.5^	***C*** mg·g^−1^	**R^2^**
$\mathrm{Nonlinear}:\;q=k_{i}t^{0.5}+C$	q vs. t	5.8358932	12.760076	0.8648146
$\mathrm{Linear}:\;q=k_{i}t^{0.5}+C$	$q\;\mathrm{vs}.\;t^{0.5}$	5.835941	12.75952	0.864815
**Pore Diffusion Model**	**Plotting**	***k_p_*** min^−0.5^	***D_ii_*** cm^2^·min^−1^	**R2**
$\frac{q_{t}}{q_{e}}=F=\frac{6}{r_{o}}{\left(-\frac{D_{ii}\cdot t}{\pi }\right)}^{1/2}=k_{p}*t^{1/2}\;$	$\frac{q_{t}}{q_{e}}\;\mathrm{vs}.\;t^{0.5}$	0.074761776	2.74 × 10^−8^	0.865
**Film Diffusion Model**	**Plotting**	***k_fd_*** min^−1^	***D_ii_*** cm^2^·min^−1^	**R2**
$\ln\left(1-{\frac{q_{t\;}}{q}}_{e}\right)=-\left(\frac{D_{i}}{r_{o}^{2}}\right)\cdot \pi^{2}\cdot t=-k_{fd}\cdot t$	$\ln\left(1-{\frac{q_{t\;}}{q}}_{e}\right)\;\mathrm{vs}.\;t$	0.019093063	1.096E−07	0.9729

**Table 7:** Analysis of raw and treated brackish water under the optimized conditions.

Sample	Parameters								
	Color	Turbidity (NTU)	Conductivity (µS/cm)	ϛ-Potential (mV)	TSS (ppm	TDS (ppm)	Na (ppm)	Ca (ppm)	K (ppm)
Raw water	Light grey	25	10,000	−25	10	10,000	8000	500	350
Treated water	Clear	5	4000	−5	4	2015	800	166	102

**Table 8:** The removal efficiency of cations from brackish water over fresh and exploited adsorbent.

Cycle	1 (Fresh Adsorbent)	2	3	4	5	6
**R %**	79.85	78.77	78.14	77.76	77.2	77.1

**Table 9:** Na^+^ values for maximum adsorption of P. australis in comparison with other adsorbents in the literature published.

Adsorbent	*q_max_* (mg/g)/R.R	Isotherms	Kinetic	Ref.
Canadian (CMZ), Bear River (BRZ), and St. Cloud (SCZ) zeolites, the application of natural and pre-treated zeolites	*q_max_* = Natural BRZ (14.3 ± 0.4 mg/g), Natural CMZ (5.8 ± 0.5 mg/g), and SCZ (5.6 ± 0.7 mg/g)			[24,37]
amorphous carbon thin film (ACTF)	NA/35	freundlich R^2^ = 0.99 n = 4.18 *K_f_* = 4.055	Pseudo (2) R^2^ = 0.99 *q_e_* = 105 *k*_2_ = 3.066	[4]
RICE HUSK CARBONACEOUS ADSORBENTS	NA/77		Intraparticle R^2^ = 0.993 *K_i_* = 3.84 *C* = −10.07 Pseudo (1) R^2^ = 0.995 *Q_e_* = 158 *K*_1_ = 0.0014	[23]
Natural zeolite (acid activated zeolite at 30%wt solid ratio) in Coal Seam Gas (CSG) waters	NA/67.55			[38]
zeolite materials			Pseudo (2) R^2^ = 0.99 *Q_e_* = 38.28 *k*_2_ = 0.002	[24]
P. australis	117.68/90	Langmuir (nonlinear)R^2^ = 0.96 *q_o_* = 189.7632 *K_L_* = 0.001245 freundlich R^2^ = 0.99 n = 1.90501 *K_f_* =2.729497	Pseudo (2) R^2^ = 0.97 *Q_e_* = 119.771 *k*_2_ = 0.000139 film diffusion model R^2^ = 0.97 *D_ii_* cm^2^min^−1^ = 1.096 × 10^−7^ *k_fd_* = 0.019093063	This work

## Data Availability

All data generated or analyzed during this study are included in this published article.

## Supplementary Materials

The following are included in the Supplementary Materials: Equation S1: Adsorption kinetics and Equation S2 Adsorption isotherms.

## Abbreviation

Fourier transform infrared	FTIR
Brunauer–Emmett–Teller	BET
Energy dispersive radiation spectroscopy	EDX
Biological oxygen demand	BOD
Chemical oxygen demand	COD
Total dissolved solids	TDS
X-ray diffraction	XRD
Reverse osmosis	(RO)
Nano-Filtration	(NF)
Electrodialysis	(ED)
Pore size distribution curve	(PSD)
