# Supplementary material for: Phragmites australis (Reed) as an Efficient, Eco-Friendly Adsorbent for Brackish Water Pre-Treatment in Reverse Osmosis: A Kinetic Study
Source: Molecules. 2021 Oct 3;26(19):6016. doi: 10.3390/molecules26196016 (PMC8512057; doi:10.3390/molecules26196016)
Supplement: Supplementary file 1 [file molecules-26-06016-s001.zip › molecules-1367713-supplementary.pdf]

1. Adsorption Kinetics

The equation gives the linear form of Lagergren's equation of pseudo-first-order model:

$$.\ln(q_e - q_t) = \ln q_e - K_1 t$$

q_e and q_t are the amounts adsorbed (mg g^{-1}) at equilibrium and time *t*, respectively, while k_1 (min^{-1}) is the rate constant of pseudo-first-order adsorption. The rate constant k_1 is detected from the slope of the plot of log $(q_e - q_t)$ versus *t*. The pseudo–the second-order equation is stated as follows:

$$\frac{t}{q_t} = \frac{1}{K_2\ q_e^2} + \frac{t}{q_t}$$

Where k_2 is the rate constant (g.mg^{-1} min^{-1}), the plot of t/q_t versus *t* gives a straight line with slope = $1/q_e$ and intercept = k_2.

2. Adsorption Isotherms

The equation gives the linear equation of this isotherm:

$$Ce/q_e = 1/q_o K_L + Ce/q_o$$

In cases where C_e is the balance of the adsorbate (mg/L), q_e shall be the amount of cations adsorbed in the balance of the adsorbent (mg/g) per g, q_o the maximum adsorption rate of the adsorbent (mg/g), and k_L shall be the maximum isotherm constant Langmuir (L/mg).

Furthermore, it is possible to estimate the dimensional separation factor constant (DDF) from Langmuir, which gives essential information on the nature of adsorption. The R_L >1 and $0 < R_L < 1$ values reflect adsorption that is both unfavourable and beneficial; the R_L =1 and the RL = 0 values show a linear and irreversible adsorption accordingly. The R_L values of Table 4 indicate that sodium biosorption is beneficial. Freundlich isotherm commonly describes the adsorption properties of heterogeneous surfaces, viz. surfaces with active sites of non-equal adsorption energies. The linear form of this isotherm is given by:

$$\ln q_e = \ln K_F + \frac{1}{n} \ln C_e$$

K_F is the Freundlich isotherm constant. An indicator of adsorption capacity and n is a dimensionless factor depending on the nature of adsorbent and adsorbate at a particular temperature. These parameters could be determined by plotting $\ln q_e$ versus $\ln C_e$. The value 1/n is a heterogeneity parameter, where 1/n is a heterogeneity parameter, and the smaller 1/n, the more heterogeneity is anticipated.
